# *Helicobacter pylori* Seropositivity: Prevalence, Associations, and the Impact on Incident Metabolic Diseases/Risk Factors in the Population-Based KORA Study

**DOI:** 10.3389/fpubh.2019.00096

**Published:** 2019-04-24

**Authors:** Nina Wawro, Ute Amann, Julia Butt, Christa Meisinger, Manas K. Akmatov, Frank Pessler, Annette Peters, Wolfgang Rathmann, Stefan Kääb, Tim Waterboer, Jakob Linseisen

**Affiliations:** ^1^German Research Center for Environmental Health (GmbH), Institute of Epidemiology II, Munich, Germany; ^2^German Center for Diabetes Research (DZD e.V.), Munich, Germany; ^3^Chair of Epidemiology, Ludwig-Maximilians-Universität München, UNIKA-T, Augsburg, Germany; ^4^German Research Center for Environmental Health (GmbH), Independent Research Group Clinical Epidemiology, Munich, Germany; ^5^Infections and Cancer Epidemiology, Infection, Inflammation and Cancer Program, German Cancer Research Center (DKFZ), Heidelberg, Germany; ^6^TWINCORE, Centre for Experimental and Clinical Infection Research, Hanover, Germany; ^7^Helmholtz-Zentrum für Infektionsforschung (HZI), Braunschweig, Germany; ^8^Deutsches Diabeteszentrum, Institute for Biometrics and Epidemiology, Düsseldorf, Germany; ^9^Medizinische Klinik und Poliklinik I, Campus Grosshadern, Ludwig-Maximilians-Universität München, Munich, Germany

**Keywords:** *Helicobacter pylori*, multiplex serology, infection, prevalence, metabolic diseases

## Abstract

**Introduction:** *Helicobacter pylori* (*H. pylori*) is a common infection and known risk factor for gastric cancer. We assessed cross-sectional and longitudinal associations to study the impact of *H. pylori* seropositivity on metabolic diseases.

**Methods:** *Helicobacter pylori* seropositivity in serum samples of the KORA study was analyzed by multiplex serology. We calculated sex-specific prevalence of *H. pylori* seropositivity for the year 2007 based on the first follow-up survey (termed F4) of the KORA study S4. We identified factors associated with *H. pylori* seropositivity in the F4 survey. Further, we assessed relative risks of incident metabolic diseases/risk factors at the time of the second follow-up survey of S4 (termed FF4) and *H. pylori* seropositivity at the F4 survey as a determinant. Models were adjusted for age, sex, overweight status, physical activity, smoking status, education level, alcohol intake, and other metabolic diseases.

**Results:** Based on 3,037 persons aged 32 to 82 years, the *H. pylori* prevalence for 2007 was 30.2% in men (*n* = 1,465) and 28.1% in women (*n* = 1,572). Increasing age, current smoking, low education and no alcohol intake were significantly associated with *H. pylori* seropositivity in the F4 survey. However, no association between *H. pylori* seropositivity and BMI, metabolic diseases (type 2 diabetes, hypertension and dyslipidemia, gout or increased uric acid) and gastrointestinal diseases (gastritis, inflammatory bowel disease, and gastric or duodenal ulcer) was observed. No significant associations between *H. pylori* seropositivity and one of the five investigated incident metabolic diseases/risk factors were detected in the longitudinal analysis.

**Conclusion:** We identified associations between age, smoking, education and alcohol intake and *H. pylori* seropositivity but no impact of *H. pylori* seropositivity on incident metabolic diseases/risk factors.

## Background

*Helicobacter pylori* (*H. pylori*) infection is one of the world's most common infections. A recent meta-analysis on global prevalence of *H. pylori* infection reported a wide range from 18.9% in Switzerland up to 87.7% in Nigeria (1). The prevalence in the African region is ~70%, the highest world-wide and is influenced by socio-economic status as well as levels of hygiene (1). As the majority of *H. pylori* infections are asymptomatic, eradication therapy is not applied to these persons and the infection persists over time, leading to inflammatory changes of the gastric mucosa. A potential risk for severe gastrointestinal diseases including gastric cancer in persons infected with *H. pylori* is known (2–5). In their recent review, Cellini et al. (6) describe the pathogenic link to be a cross-reactivity mechanism of the *H. pylori* infection that induces proliferation of CD4^+^ T lymphocytes. These are the lymphocytes that recognize epitopes of *H. pylori*, which are structurally similar to those of H+/K+-ATPase (7). In the absence of peripheral tolerance, a Th1-driven autoreactive clone is activated (8) when dendritic cells present these shared epitopes to naïve T cells. Besides gastric cancer, *H. pylori* infection has been suggested to be causally linked to a variety of extra-gastric diseases and metabolic derangements (3, 9). In particular, the impact of *H. pylori* seropositivity on newly developed diabetes mellitus has been studied over the last years providing conflicting results. A prospective study over 10 years including Latino elderlies living in California found a positive association between seropositive *H. pylori* status and incident diabetes (10). However, Yu et al. reported an impact of gastric atrophy but not of *H. pylori* infection on the incidence of diabetes (11). Further, the influence of *H. pylori* infection on the incidence of other metabolic disorders, gastrointestinal diseases without gastric cancer and as well extra-gastric diseases, such as neurological or hematological diseases, asthma, allergies, immune disorders or skin diseases, has been studied widely (3, 9, 12). Lastly, there is a large potential burden of impaired drug absorption in the stomach, as persistent *H. pylori* infections can possibly lead to an altered gastric pH (13). The aim of our study was to calculate the sex-specific prevalence of *H. pylori* seropositivity for the year 2007 based on the follow-up survey F4 of the KORA S4 study. Secondly, in a cross-sectional approach we aimed to investigate associations between *H. pylori* seropositivity at F4 and lifestyle factors, metabolic, gastrointestinal, and other diseases. Finally, in a prospective design we investigated the association between seropositive *H. pylori* status at F4 and incident metabolic diseases/risk factors at FF4 (second follow-up of S4) defined as type 2 diabetes, obesity, relative weight change increase, hypertension, and dyslipidemia.

## Subjects and Methods

Our analyses are based on data from the Cooperative Health Research in the Region of Augsburg (KORA) F4 study (2006–2008) and the KORA FF4 study (2013–2014). They are both follow-up examinations of the population-based KORA S4 study (1999–2001), conducted in the city of Augsburg and two surrounding counties. The design and background of the KORA studies have been described in detail elsewhere (14). In brief, medical examinations by trained staff, self-administered questionnaires and the collection of biosamples were applied within the studies. Details on the assessment of the variables of interest are given below. The investigations were carried out in accordance with the Declaration of Helsinki, including written informed consent from all participants. The study was approved by the ethics committee of the Bavarian Chamber of Physicians (Munich, Germany).

The data set included 3,080 participants at F4. Thereof, 3,037 participants had information on their *H. pylori* status at F4 available, but not necessarily at FF4. A subset of 2,075 persons participated in both surveys (F4 and FF4) and had their *H. pylori* status at both survey time-points available. Prevalence of *H. pylori* seropositivity and its association with certain diseases and lifestyle factors was described for participants of the F4 survey. We explored the impact of acute and past *H. pylori* infection on incident diseases/risk factors in the longitudinal design with F4 as baseline and FF4 as follow-up examination to identify incident diseases.

### Definition of Variables and Assessment

Antibody responses to *H. pylori* were determined by multiplex serology as described elsewhere (15). Briefly, 15 *H. pylori* proteins were recombinantly expressed as GST-X-tag fusion proteins and each antigen affinity-purified on distinct fluorescently labeled glutathione-casein coated bead sets (SeroMap, Luminex Corp., Austin, Tx, USA). A mix of these differently labeled, antigen-coupled bead sets was incubated with serum. A Luminex xMAP analyzer (Luminex Corp., Austin, Tx, USA) identifies the bead set and simultaneously quantifies bound serum IgA, IgM, and IgG by a reporter fluorescent conjugate, Streptavidin-R-Phycoerythrin. The level of antibody response is given as median fluorescence intensity (MFI). Antigen specific cut-offs were applied and seropositivity to *H. pylori* defined as being positive to at least 4 antigens.

Outcomes of interest for the longitudinal analysis are the following newly developed metabolic diseases/risk factors: type 2 diabetes, obesity, relative weight change increase, hypertension, and dyslipidemia. These variables were assessed at F4 and at FF4 as described subsequently. Prevalent cases at F4 were excluded from the respective longitudinal association analysis of *H. pylori* seropositivity at F4 and incident disease/risk factor at FF4.

Type 2 diabetes was assessed at F4 and FF4 based on an oral glucose tolerance (OGT) test and the WHO definition and classification of diabetes mellitus (16), and/or diabetes medication reported and further validated by hospital records or by contacting the participants' treating physicians. Therefore, this variable contains known validated diabetic participants (without further OGT test) as well as newly diagnosed type 2 diabetic participants identified by the OGT test. Participants with type 1 diabetes (*n* = 6 at F4) or unclear diabetes status (*n* = 38 at F4) were discarded from the analysis.

Obese participants were identified at F4 and FF4 by a body mass index (BMI) ≥30 kg/m^2^. The BMI was derived from body weight and height and expressed in kg/m^2^. Additionally, the relative weight change between F4 and FF4 was calculated as the difference of both weights divided by the weight at F4. Relative weight change increase was then defined as a relative weight change greater than zero (yes/no).

Hypertensive participants were identified at F4 and FF4 by their “actual hypertensive” status (yes/no). Actual hypertensive participants were identified according to WHO-ISH guidelines (17) as either normotensive, aware and treated, or hypertensive and aware (treated or not) or hypertensive and unaware.

Dyslipidemia at F4 and FF4 was defined by laboratory data as a ratio of total cholesterol to HDL cholesterol greater or equal to five.

### Definition of Covariates

All analyses were adjusted for age, sex, overweight status based on BMI (if not used as outcome variable), smoking status, education level, physical activity and alcohol intake. Age was included as continuous variable. Smoking was categorized into current, former and never smokers. The education level was available as low, medium and high representing “Hauptschule / Volksschule,” “Mittlere Reife / Realschule,” and “Abitur / Fachabitur / Fachhochschulreife,” respectively. Alcohol intake was coded as none, medium (up to 40 g/day) and high (more than 40 g/day) based on an already categorized alcohol intake variable. Participants were classified as physically inactive when they reported to do sport activities irregularly or <1 h per week in both seasons and as physical active otherwise.

The following variables were included in the descriptive analysis and the cross-sectional analysis: continuous BMI, known gout or increased serum uric acid concentration (the corresponding question assessed a physician-based diagnosis during the last 12 months), known gastritis (last 12 months), known inflammatory bowel disease (last 12 months) gastric or duodenal ulcer (last 12 months). In addition, information on the following laboratory data was included: total cholesterol (mmol/L), fasting plasma glucose (mmol/L), and cytotoxin-associated gene-A (CagA) positivity (>1,800 MFI).

### Statistical Analysis

A descriptive analysis of all participants who had their HP status available at F4 and FF4 (*n* = 2,075) was carried out. We report absolute and relative numbers for all adjustment variables and clinical characteristics of interest that had been described above. Further, means and standard deviations (SD) are given for the laboratory data. To compare the groups defined by *H. pylori* seropositivity the Wilcoxon rank sum test for continuous variables and the chi^2^-test for categorical variables were applied.

To describe the prevalence of acute and past *H. pylori* infection, age-standardized proportions were calculated for the KORA F4 population. As a reference group, the age distribution in Germany in the year 2007 was used, split into 10 year intervals (18). All participants with available *H. pylori* status were included in the analyses at F4, resulting in *n* = 3,037.

A cross-sectional approach was applied to identify associations of *H. pylori* seropositivity at F4. In the logistic regression model, all covariates and clinical outcomes described above were included. The odds ratio (OR) and 95% confidence interval (CI) are reported.

In a longitudinal design, we aimed to identify associations of *H. pylori* seropositivity at the F4 survey and incident metabolic diseases/risk factors at FF4. As mentioned before, prevalent cases at F4 were excluded from the respective analysis. For each outcome we fitted three generalized regression models using the log-link function to estimate relative risks (RR). Model 1 represents a raw model, adjusting only for age and sex. Model 2 additionally included obesity, physical activity, smoking status, education level, and alcohol consumption as covariates. Model 3 is a fully adjusted model, including also information on hypertension, dyslipidemia, type 2 diabetes and gout or increased uric acid. When investigating newly developed obesity, relative weight change increase, hypertension, dyslipidemia and type 2 diabetes, the corresponding adjustment variable was excluded in models 2 and 3. All adjustment information was taken from the F4 survey. Effect modification by age and sex was explored.

As a sensitivity analysis for diabetes, not only were participants suffering from type 2 diabetes at F4 excluded, but also those who were identified as pre-diabetic by the OGT test applied at the F4 examination (16). Generalized regression models 1, 2, and 3 were fitted. Further, analogous linear models with fasting plasma glucose levels as continuous outcome variable were fitted as sensitivity analyses.

All statistical analyses were performed using R3.3.2.

## Results

### Study Population

The mean age in the full data set used for calculating the prevalence was 56.2 years (SD 13.3) at F4 (*n* = 3,037). Mean age was 56.7 years (SD 13.4) in men and 55.6 years (SD 13.1) in women (*p* < 0.05).

Table 1 shows the baseline characteristics of the 2,075 participants of both surveys stratified by *H. pylori* seropositivity status at the F4 survey. The 586 (28.2%) persons with acute or past *H. pylori* infection were significantly (*p* < 0.05) older, less educated, had higher BMI, suffered more often from prediabetes/diabetes type 2, hypertension, gout or increased uric acid, and showed differences in alcohol intake compared with their counterparts. Conversely, no differences were observed in the prevalence of gastritis, inflammatory bowel disease, gastric or duodenal ulcer and dyslipidemia. In addition, the groups did not differ by sex, physical activity, smoking, total cholesterol, and fasting plasma glucose level. Sixty-three percent of the *H. pylori*-positive participants were also CagA-positive.

**Table 1:** Frequency (%) and mean (SD) of characteristic variables by *H. pylori* seropositivity status at the F4 survey in persons who participated in both surveys (*n* = 2,075).

		***H. pylori*** **Seropositivity**		
	**Sample size n**	**Negative (*n* = 1,489)**	**Positive (*n* = 586)**	*p*-value^*^
**SOCIODEMOGRAPHIC CHARACTERISTICS**				
Age (years)	2,075	52.3 (12.0)	58.2 (11.9)	**<0.0001**
Sex	2,075			0.33
Men		710 (0.48)	294 (0.50)	
Women		779 (0.52)	292 (0.50)	
Education	2,070			**<0.0001**
Low		661 (0.44)	352 (0.60)	
Medium		411 (0.27)	115 (0.20)	
High		416 (0.28)	115 (0.20)	
BMI (kg/m^2^)	2,067	27.0 (4.6)	27.9 (4.7)	**<0.0001**
Obesity (BMI ≥ 30 kg/m^2^)	2,067			**0.0005**
Yes		323 (0.22)	171 (0.29)	
No		1,158 (0.78)	415 (0.71)	
Smoking	2,075			0.55
Current		245 (0.16)	102 (0.17)	
Former		567 (0.38)	233 (0.40)	
Never		677 (0.45)	251 (0.43)	
Alcohol intake	2,075			**<0.0001**
No		385 (0.26)	201 (0.34)	
Medium (>0– < 40 g/day)		931 (0.62)	315 (0.54)	
High (40–>80 g/day)		173 (0.11)	70 (0.12)	
Physical activity	2,075			0.13
Active		879 (0.59)	324 (0.55)	
Inactive		610 (0.41)	262 (0.45)	
**CLINICAL CHARACTERISTICS**				
Diabetes	2,075			**0.0002^a^**
No diabetes		1,155 (0.78)	410 (0.70)	
Prediabetes		196 (0.13)	100 (0.17)	
Type 2 Diabetes		104 (0.07)	66 (0.11)	
Other diabetes type		6 (0.00)	0 (0.00)	
Unknown		28 (0.02)	10 (0.02)	
Hypertension	2,073			**<0.0001**
Yes		435 (0.29)	241 (0.41)	
No		1052 (0.71)	345 (0.59)	
Dyslipidemia	2,074			0.65
Yes		289 (0.19)	108 (0.18)	
No		1199 (0.81)	478 (0.82)	
Gout or increased uric acid	1,875			**0.02**
Yes		61 (0.04)	33 (0.07)	
No		1254 (0.90)	419 (0.86)	
Unknown		72 (0.05)	36 (0.07)	
Gastritis	1,880			0.082
Yes		88 (0.06)	31 (0.06)	
No		1248 (0.90)	443 (0.90)	
Unknown		54 (0.04)	16 (0.03)	
Inflammatory bowel disease	1,879			0.415
Yes		34 (0.02)	15 (0.03)	
No		1319 (0.95)	459 (0.93)	
Unknown		35 (0.03)	17 (0.03)	
Gastric or duodenal ulcer	1,875			0.678
Yes		14 (0.01)	6 (0.01)	
No		1341 (0.97)	469 (0.96)	
Unknown		31 (0.02)	14 (0.03)	
**LABORATORY DATA/SEROLOGY**				
Total cholesterol (mmol/L)	2,075	215.5 (38.9)	214.6 (37.0)	0.552
Fasting plasma glucose (mmol/L)	2,062	96.4 (16.6)	97.4 (16.0)	0.053
CagA positive (>1800 MFI)	2,075	111 (0.07)	372 (0.63)	**<0.0001**

* *p-value calculated with Wilcoxon rank sum test for continuous variables or chi^2^-test for categorical variables. p-values < 0.05 are marked bold*.

a *p-value calculated without subgroups smaller than 10 persons*.

### Age-Standardized Prevalence of *H. pylori* Seropositivity

Based on the 3,037 persons aged between 32 and 82 years who participated at the F4 survey, the age-standardized *H. pylori* prevalence for the year 2007 was 30.2% in men (*n* = 1,465) and 28.1% in women (*n* = 1,572). When examining the 2,075 participants with information on their *H. pylori* status available in both surveys, we found 73 participants who were *H. pylori* seropositive at the F4 survey, but not at the FF4 survey. On the contrary, we observed 72 incident cases of *H. pylori* infection at the FF4 survey.

### Variables Associated With a *H. pylori* Seropositivity at the F4 Survey

In the multivariable logistic regression analysis including 2,458 persons with complete covariate data available at F4, the following factors were significantly associated with a seropositive *H. pylori* status: increasing age, current smoking, low education level compared to medium education level and no alcohol intake. In this cross-sectional design adjusted for co-variables at the time of the F4 survey, no associations between metabolic diseases/risk factors (BMI, type 2 diabetes, hypertension and dyslipidemia, gout or increased uric acid) and gastrointestinal disease (gastritis, inflammatory bowel disease, gastric, or duodenal ulcer) were observed (Table 2).

**Table 2:** Variables associated with *H. pylori* seropositivity at the F4 survey.

Study population (*n* = 2,458)^*^	**OR (95% CI)**	*p*-value^**^
Sex (women vs. men)	0.88 (0.72–1.08)	0.21
**Age (years)**	1.04 (1.03–1.05)	**<0.0001**
Body mass index (kg/m^2^)	1.00 (0.98–1.02)	0.84
Physical activity (inactive vs. active)	1.18 (0.97–1.42)	0.09
**Smoking (former vs. current)**	0.75 (0.58–0.97)	**0.025**
**Smoking (never vs. current)**	0.67 (0.52–0.87)	**0.002**
**Education (medium vs. low)**	0.75 (0.60–0.95)	**0.016**
Education (high vs. low)	0.78 (0.61–1.00)	0.051
**Alcohol intake (medium vs. no)**	0.73 (0.59–0.90)	**0.003**
**Alcohol intake (high vs. no)**	0.72 (0.52–0.99)	**0.046**
Hypertension (no vs. yes)	0.87 (0.70–1.08)	0.20
Dyslipidemia (no vs. yes)	1.04 (0.83–1.32)	0.72
Type 2 diabetes (no vs. yes)	0.93 (0.67–1.28)	0.65
Gout or increased uric acid (no vs. yes)	0.85 (0.58–1.27)	0.43
Gout or increased uric acid (do not know vs. yes)	0.97 (0.58–1.64)	0.92
Gastritis (no vs. yes)	0.92 (0.64–1.35)	0.67
Gastritis (do not know vs. yes)	0.86 (0.46–1.59)	0.63
Gastric or duodenal ulcer (no vs. yes)	1.01 (0.43–2.48)	0.99
Gastric or duodenal ulcer (do not know vs. yes)	0.92 (0.32–2.70)	0.87
Inflammatory bowel disease (no vs. yes)	1.14 (0.65–2.07)	0.66
Inflammatory bowel disease (do not know vs. yes)	1.13 (0.49–2.60)	0.78

** *p-values < 0.05 are marked bold*.

* *n = 579 persons deleted due to missing values*.

### Associations of Incident Metabolic Diseases and *H. pylori* Seropositivity

Table 3 provides an overview of the incident metabolic diseases/risk factors used as outcome variables and their frequencies in the 2,075 subjects participating in both surveys. In the generalized regression model analyses, no significant associations between *H. pylori* seropositivity at the time of the F4 survey and incident metabolic diseases/risk factors at time of the FF4 survey were observed. Sample size, adjusted relative risks (RR) and 95% CI of every statistical analysis are presented in Tables 4, 5. Inclusion of CagA seropositivity in the models did not change the results (data not shown). For the outcome “incident diabetes,” we ran a sensitivity analysis by additionally excluding participants with pre-diabetic status at F4 from the analysis. This revealed a non-significant increased risk of newly developed diabetes (model 3: OR 1.93, *p* = 0.056) in participants with acute or past *H. pylori* infection (Table 5). Further sensitivity analysis on fasting plasma glucose levels led to similar results (data not shown). Adding the interaction effects with *H. pylori* seropositivity to regression model 1 in the longitudinal analyses did not show effect modification by age and sex (data not shown).

**Table 3:** Metabolic diseases/risk factors defined as outcome variables and their frequencies in participants assessed at the FF4 survey.

**Outcome variables**	**Assessment at the FF4 survey**			Sample size for regression analysis^*^
	**Yes**	**No**	**Not known**	
Relative weight change increase	1154	913	8	2,067
Incident type 2 diabetes	1,670	130	99	1,800
Incident hypertension	227	1,168	4	1,395
Incident dyslipidemia	216	1,857	2	2,073
Incident obesity	137	1,436	8	1,571

* *Sample size varies by outcome variable due to different number of excluded persons who suffered already from the disease at F4, or reported at F4 and/or at FF4 that they do not know about the disease*.

**Table 4:** Association of *H. pylori* seropositivity with incident hypertension, obesity, and relative weight change increase.

**Analysis**	**Incident hypertension (yes/no)**			**Incident obesity (yes/no)**			**Relative weight change increase (yes/no)**		
	Sample size^*^	**RR (95% CI)**	***p*-value**	Sample size^*^	**RR (95% CI)**	***p*-value**	Sample size^*^	**RR (95% CI)**	***p*-value**
Model 1^a^	1,395	1.15 (0.90;1.47)	0.28	1,572	0.97 (0.66;1.40)	0.89	2,067	1.02 (0.93;1.11)	0.73
Model 2^b^	1,385	1.02 (0.81;1.28)	0.87	1,569	0.90 (0.61;1.31)	0.60	2,062	1.02 (0.93;1.12)	0.61
Model 3^c^	1,297	1.10 (0.86;1.42)	0.44	1,405	0.96 (0.63;1.41)	0.84	1,825	1.03 (0.94; 1.13)	0.46

* *Sample sizes of the models were based on complete case for outcome variables and respective co-variables*.

a *Adjusted for age (cont.) and sex*.

b *Additional adjustment for physical activity, education, alcohol intake, smoking, and obesity (not for the outcomes “incident obesity” and “relative weight change increase”)*.

c *Additional adjustment for all other clinical characteristics (diabetes, dyslipidemia, hypertension, and gout or increased uric acid levels) with assessment at F4*.

**Table 5:** Association of *H. pylori* seropositivity with incident type 2 diabetes and dyslipidemia.

**Analysis**	**Incident type 2 diabetes**						**Incident dyslipidemia**		
	**In persons without type 2 diabetes at F4**			**In persons without type 2 diabetes or prediabetes at F4**			
	Sample size^*^	**RR (95% CI)**	***p*-value**	Sample size^*^	**RR (95% CI)**	***p*-value**	Sample size^*^	**RR (95% CI)**	***p*-value**
Model 1^a^	1,800	1.07 (0.76;1.50)	0.69	1,513	1.77 (0.92;3.33)	0.08	2,073	0.91 (0.67;1.21)	0.53
Model 2^b^	1,795	0.93 (0.66;1.30)	0.65	1,508	1.55 (0.81;2.94)	0.18	2,060	0.82 (0.61;1.09)	0.18
Model 3^c^	1,646	1.08 (0.76;1.52)	0.68	1,411	1.93 (0.98;3.61)	0.06	1,824	0.85 (0.62;1.14)	0.28

a *Adjusted for age (cont.) and sex*.

b *Additional adjustment for obesity, physical activity, education, alcohol intake, and smoking*.

c *Additional adjustment for the baseline variables hypertension, gout, or increased uric acid blood levels, and diabetes or dyslipidemia (depending on outcome parameter)*.

* *Sample sizes of the models were based on complete case for outcome variables and respective co-variables*.

## Discussion

Our results from the population-based KORA study are in line with earlier data on the prevalence of *H. pylori* seropositivity in West Germany. Further, we confirmed age, education level, smoking and alcohol intake to be associated with *H. pylori* seropositivity. The longitudinal analysis showed no significant association between *H. pylori* seropositivity and incident metabolic diseases/risk factors.

Our finding on the age-standardized *H. pylori* prevalence of 30% in men and 28% in women for the year 2007 is in accordance with an earlier study conducted in West Germany (19) using *H. pylori* serology. However, using the same assay in a population-based study conducted in the 80s in Germany, Michel, Pawlita (2) reported a *H. pylori* prevalence of 49% in men and 48% in women. A recent systematic review and meta-analysis on global *H. pylori* prevalence reported a pooled prevalence estimate for Germany of 35.3% (95% CI 31.2–39.4) based on eight studies which were conducted between 1987 and 2010 (1). Six out of these eight German studies used also serology as method to diagnose *H. pylori* infection and revealed prevalence estimates from 31.7% (19) up to 44.4% (20), depending on study period and region. It is known from previous studies that *H. pylori* prevalence depends on the development status of a country, the study region within one country, and socioeconomic status of the participants, see e.g. (21). For Germany, it is known that depending on the selected areas and study period the prevalence varies accordingly (22). Prevalence was significantly higher in all age groups of the eastern part of Germany compared to West Germany (19, 20, 22). In addition, a decrease in *H. pylori* prevalence over time in many developed countries was described (2, 21).

Our results confirmed that age, education level and alcohol intake are associated with *H. pylori* seropositivity, but lipid levels are not (23). In contrast to the findings of Rosenstock et al. (23), smoking is significantly associated with *H.pylori* seropositivity in our analysis whereas BMI is not. In addition, we observed no association for type 2 diabetes, hypertension and dyslipidemia, which is in accordance with the results from earlier cross-sectional studies, see e.g. (23, 24). Age, sex, BMI, and education level have been reported to be related to *H. pylori* infection earlier (25), as well as an inverse association with alcohol consumption (26) and, among other factors, socioeconomic status (22). Interestingly we did not find an association between gastro-intestinal diseases such as gastritis and gastric or duodenal ulcer and *H. pylori* seropositivity. One explanation might be that our population includes a high number of symptom-free and/or successfully treated *H. pylori* infections.

An explanation for the association of alcohol intake and *H. pylori* seropositivity might be that besides the bactericidal effect, alcoholic beverages stimulate gastric acid secretion. This could result in lowering the pH in the stomach, which could impact the *H. pylori* load (27, 28). Regarding the association of smoking and *H. pylori* infection, Endoh and Leung (29) suggested that nicotine reduces gastric mucosal blood flow, epidermal growth factor secretion, and mucus secretion, thus facilitating *H. pylori* colonization (30). Not every *H. pylori* infected participant will develop gastric atrophy (11). This aspect could also have influenced our results in the longitudinal analysis, where we did not observe a significant association of *H. pylori* seropositivity and incident metabolic diseases/risk factors. *H. pylori* infections can be seen as the most important cause of gastritis and the development of gastric and duodenal ulcer (31). A reason for the non-associations found in our longitudinal analysis could be the relatively short follow-up-time of 6–9 years between the two surveys. Therefore, a seropositive *H. pylori* status in the participants without significantly higher frequencies of gastrointestinal risk factors such as gastritis or peptic ulcer disease at baseline (F4 survey) might need longer periods and cofactors to evolve non-gastrointestinal diseases due to inflammatory processes suggested in the pathogenesis of those diseases (21). Although the frequency of CagA-positive strains within the *H. pylori* infected patients in our study was higher (63%) compared with an earlier study in Eastern Germany (32), inclusion of this virulence factor did not change the present results. Earlier prospective cohort studies regarding the impact of *H. pylori* infection on the incidence of diabetes reported contradictory results (10, 11). In addition, a recent meta-analysis reported increased risks of type 1 and type 2 diabetes mellitus with *H. pylori* infection for the included 17 case-control studies, but no significance was observed for the 2 included cohort studies (33). As a longitudinal design is mandatory for investigating incident cases without the limitation of potential “reverse causality,” our prospective study adds further non-significant findings about a potential association between *H. pylori* seropositivity and incident diabetes. To answer the conflicting results on diabetes risk, further prospective studies might be necessary in participants without prevalent diabetes or prediabetes. The diagnosis of an *H. pylori* infection should also be based on histology obtained by endoscopy to identify all cases. Furthermore, an association between *H. pylori* infection and newly developed obesity, hypertension, dyslipidemia or relative weight change increase was not observed in our study. As previous studies investigating cardiovascular diseases (9, 34), hypertension (35), lipid profile (3, 35–37), obesity (3), and BMI (35, 36, 38) have been mainly cross-sectional or ecological, rather than prospective in design, little evidence exists so far for meaningful causative associations.

Because our results are based on a representative sample of the German population, plausible estimates of current *H. pylori* prevalence could be reported. Age, education level, smoking and alcohol intake were associated with *H. pylori* seropositivity. Based on the findings of missing associations between acute or past *H. pylori* infection and incident metabolic diseases/risk factors, we conclude that further prospective studies covering a sufficient time span along with histological confirmation of active *H. pylori* infection are required.

## Ethics Statement

The investigations were carried out in accordance with the Declaration of Helsinki, including written informed consent from all participants. The study was approved by the ethics committee of the Bavarian Chamber of Physicians (Munich, Germany).

## Author Contributions

NW and UA conceived the study and drafted the manuscript. NW performed the statistical analyses. CM, TW, JB, FP, MA, AP, WR, SK, and JL contributed to data acquisition and to the design of the study, and critically revised the manuscript. All authors have read and approved the final manuscript.

### Conflict of Interest Statement

The authors declare that the research was conducted in the absence of any commercial or financial relationships that could be construed as a potential conflict of interest.

## Abbreviations

*H. pylori*: helicobacter pylori
BMI: Body mass index
OGT: oral glucose tolerance
OR: odds ratio
CI: confidence interval
nmol: nanomol
L: liter
MFI: median fluorescence intensity
CagA: cytotoxin-associated gene-A
KORA: Cooperative Health Research in the Region Augsburg)
S4: KORA Survey 4
F4: KORA Follow-up of S4
FF4: KORA Second Follow-up of S4.
